# Rapid and Accurate Shape-Sensing Method Using a Multi-Core Fiber Bragg Grating-Based Optical Fiber

**DOI:** 10.3390/s25144494

**Published:** 2025-07-19

**Authors:** Georgios Violakis, Nikolaos Vardakis, Zhenyu Zhang, Martin Angelmahr, Panagiotis Polygerinos

**Affiliations:** 1 School of Electrical and Computer Engineering, Hellenic Mediterranean University, Estavromenos, 71410 Heraklion, Greece; 2 Department of Fiber Optical Sensor Systems, Fraunhofer Heinrich Hertz Institute, Am Stollen 19H, 38640 Goslar, Germany; zhenyu.zhang@hhi.fraunhofer.de (Z.Z.); martin.angelmahr@hhi.fraunhofer.de (M.A.); 3 Control Systems and Robotics Laboratory, Department of Mechanical Engineering, School of Engineering, Hellenic Mediterranean University, 71004 Heraklion, Greece; polygerinos@hmu.gr

**Keywords:** optical fibers, multi-core optical fibers, shape sensing, shape reconstruction

## Abstract

**Highlights:**

**What are the main findings?**

- Novel analytical algorithm for multi-core FBG-based shape sensing using second-order polynomials;
- Experimental validation, with shape-sensing fiber tip-positioning accuracy better than 2.5%.

**What is the implication of the main finding?**

- Low computational requirements for high-speed and high-accuracy algorithm;
- Scalable method to multiple curvature-sensing nodes, ideal for high accuracy, short length (<5 m) navigation, or surface mapping applications.

**Abstract:**

Shape-sensing optical fibers have become increasingly important in applications requiring flexible navigation, spatial awareness, and deformation monitoring. Fiber Bragg Grating (FBG) sensors inscribed in multi-core optical fibers have been democratized over the years and nowadays offer a compact and robust platform for shape reconstruction. In this work, we propose a novel, computationally efficient method for determining the 3D tip position of a bent multi-core FBG-based optical fiber using a second-order polynomial approximation of the fiber’s shape. The method begins with a calibration procedure, where polynomial coefficients are fitted for known bend configurations and subsequently modeled as a function of curvature using exponential decay functions. This allows for real-time estimation of the fiber tip position from curvature measurements alone, with no need for iterative numerical solutions or high processing power. The method was validated using miniaturized test structures and achieved sub-millimeter accuracy (<0.1 mm) over a 4.5 mm displacement range. Its simplicity and accuracy make it suitable for embedded or edge-computing applications in confined navigation, structural inspection, and medical robotics.

## 1. Introduction

Shape-sensing technologies are essential for a wide range of applications, including minimally invasive surgery [1], mining exploration [2], robotics [3,4], structural health monitoring [5], and aerospace systems [6,7]. These systems enable real-time spatial awareness, deformation tracking, and closed-loop control, especially in environments with constrained or inaccessible spaces.

Shape sensors can be broadly classified into Conventional Shape Sensors (CSSs) and Fiber Optic Shape Sensors (FOSSs) [8]. CSS technologies, such as cameras, radar, and LiDAR [9,10], are non-contact and often rely on a line of sight, which limits their applicability in occluded or embedded environments. FOSSs, on the other hand, use embedded optical fibers that directly respond to strain and deformation, offering superior integration and immunity to electromagnetic interference. Examples of such applications are embedding fiber optic shape sensors in flexible materials for shape monitoring and reconstruction in real time, medical navigation in minimally invasive surgery, voids reconstruction in the mining industry, and robotic arm positioning, among many others. In the work of Floris et al., an overview of several shape-sensing technologies is presented with a special mention and timeline of development of several optical fiber shape sensors [11].

Fiber Optic Shape Sensing itself is mainly divided into two large sensor families: distributed and quasi-distributed optical fiber sensors. The first are mainly based in the detection of Raman, Rayleigh, or Brillouin scattering. Rayleigh scattering-based sensors can be split into two main technologies: Optical Time Domain Reflectometry (OTDR) and Optical Frequency Domain Reflectometry (OFDR) [12]. The latter exhibits excellent spatial resolution and dynamic range, especially when using nonlinear effects or optical backscattered reflectometry [13], at the cost of more costly and complex readout units [14], while the former is typically limited by the light source bandwidth. Brillouin scattering methods suffer both from high implementation costs, mainly attributed to the sensor readout electronics, as well as foundational limitations in shape reconstruction, such as sensitivity to light intensity variations from extrinsic factors, optical fiber placement, and mounting material, among many other factors, but are suitable for applications requiring long sensing lengths [15,16]. Recently, LED incorporation in elastic lightguides has also been used as a quasi-distributed shape-sensing system [17].

On the other hand, quasi-distributed fiber optic shape sensors are based mainly on the inscription of Fiber Bragg Grating (FBG) point sensors on multi-core optical fibers [18], a technology demonstrated back in 2000 that still remains largely popular, as FBGs are the most commercially successful optical fiber sensors and enjoy a very wide acceptance in several application fields [19,20,21,22].

The widespread use of FBGs in multi-core optical fibers for shape-sensing applications has also raised the challenge of converting the wavelength shifts provided by the FBG sensors at each sensing point into solid angles and from there into Cartesian coordinates or the shape of the optical fiber cable itself. In the work of Sefati et al., optical trackers and cameras were used in order to estimate tip position [23]. Alambeigi et al. introduced a stochastic filtering algorithm that does not require a priori calibration of the flexible end-effector but has only been validated and applied in ‘C-shaped’ deformations, while applicability and accuracy in ‘S-shaped’ deformations is yet to be evaluated [24]. Wang et al. used a shape reconstruction algorithm based on piecewise constant curvature and torsion, achieving satisfactory results, albeit with some pronounced error propagation due to both algorithmic weaknesses and manufacturing imperfections [25]. In another study, Roesthuis et al. used 3D kinematics-based and mechanics-based models for predicting deflection, with a maximum error of 3.77 mm for a 172 mm long needle with embedded FBG sensors (~2.2% error) [26]. Yi et al. converted the Bragg wavelength shifts into a local node curvature and from there applied differential geometry for shape reconstruction [27]. More recently, Lu et al. developed a 3D shape reconstruction algorithm for multi-core optical fibers with FBGs, integrating optical fiber curvature and twist information in conjunction with a local mοving average approach to enhance estimation accuracy in flexible endoscopic surgery [28]. Henken et al. used the Frenet–Serret equation of the Euler integral to reconstruct the shape of a needle tip with embedded FBG optical fibers. At a maximum deflection of 12.5 mm, the reconstruction error was 0.89 mm (~7.1%) [29]. In a later work, Chen et al. used the Frenet coordinate system as well but utilized a two-step process to improve terminal positioning errors to values below 3% [30]. More recently, neural networks were employed for multi-core optical fibers with FBGs, reaching positioning accuracies less than 1% [31]. A recent review by Zhang et al. summarizes FBGs in multi-core optical fibers and their sensing applications [32]. The above approaches highlight the diversity of approaches towards FBG-based optical fiber shape reconstruction, as currently, each approach poses its own advantages and disadvantages, with no globally accepted golden standard method.

Despite advances, a key challenge remains: accurately converting measured wavelength shifts into spatial coordinates, particularly at the fiber tip. As already mentioned, existing methods involve complex differential geometry stochastic filtering or kinematics-based reconstruction, which often require intensive computation or prior calibration using external tracking systems.

To address this, we present a new algorithm that models the bent fiber shape using a second-order polynomial fitting of the curved optical fiber shape. The method is more general and can be applied to other multi-core optical fiber configurations and provides high-accuracy results for the tip position determination. In the proposed algorithmic approach, an initial calibration takes place in planar Cartesian coordinates, and the experimentally derived values are used as the ground truth for the curve-fitting algorithm. The Bragg wavelength shift of the four-core optical fiber is constantly monitored to provide the optical fiber curvature information, and the algorithm uses this input to calculate a 2-dimensional shape reconstruction of the optical fiber as well as the optical fiber orientation in space, resulting in a full 3-dimensional coordinate set for the tip position.

## 2. Materials and Methods

### 2.1. Multi-Core Fiber and FBG Sensor Fabrication

**Figure 1:**
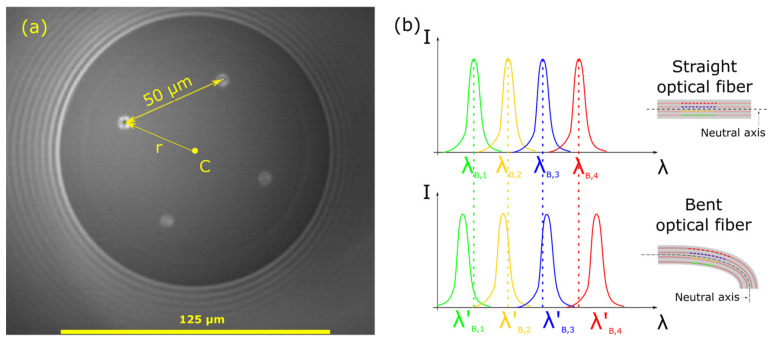
(**a**) Microscopic image of the bend-sensitive 4-core SM-4C1500 optical fiber. (**b**) Bend-sensing principle in multi-core optical fibers with FBGs inscribed in their cores.

In this work, a commercially available four-core optical fiber was used (SM-4C1500(8.0/125)/001, FiberCore Ltd., Southampton, UK) to inscribe four individual FBGs on each core in order to function as a directional bend-sensitive optical fiber. The optical fiber had a square core lattice and a core-to-core spacing of 50 μm, as illustrated in the microscopic image of this optical fiber cross-section in Figure 1a. Four individual Fiber Bragg Gratings of different central Bragg wavelengths (λ_Β_) are inscribed inside the optical fiber core by utilizing the point-by-point technique. In this technique, the FBGs are fabricated by laterally exposing the core of the fiber to a focused femtosecond laser beam, creating a single fringe at a time and scanning along the fiber length to create the FBG structure. The four FBGs are centered around the same longitudinal position along the optical fiber length in order to create a local bend-sensitive node, as shown in the upper part of Figure 1b. The total length of each FBG was 4 mm, with a reflectivity of ~40%, which was chosen as a good combination of the photo-induced refractive index and grating length that yields a satisfactory FBG spectral shape that can be easily tracked, even under large optical fiber bend angles.

The bend-sensing principle is typical for multi-core optical fibers with FBGs inscribed, as illustrated in Figure 1b. The 4 FBGs that are located at the same longitudinal optical fiber coordinate undergo different amounts of elongation or compression depending on the bend angle magnitude and direction. This physical deformation of the optical fiber is manifested as Bragg wavelength shifts Δ*λ_B_*, which are, in turn, translated into a curvature vector ***κ***, as already extensively covered in the literature [1,33]. The curvature vector is then converted into an optical fiber shape by various different methods such as solution of the Frenet–Serret differential equations [1,34], the solution of the parallel transport plane differential equations [35], or by the use of transformation matrices [36].

### 2.2. Experimental Setup and Tip Position Calibration

**Figure 2:**
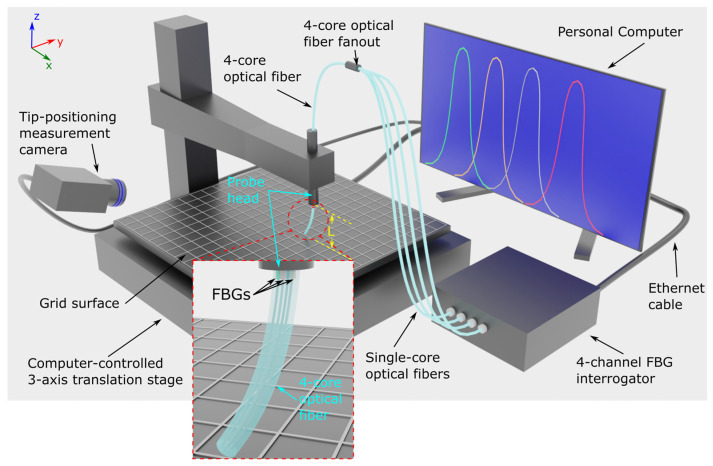
Setup for experimental determination of bend-sensitive optical fiber tip coordinates, FBG spectral read-out, and experimental evaluation of the tip coordinate evaluation algorithm.

In the present work, the curvature vector ***κ*** is calculated from the Bragg wavelength shifts of the four-core optical fiber in order to determine the tip position of a bend-sensitive optical fiber protruding from a probe, using a set of experimentally measured tip position values as the initial calibration values. The setup for the experimental measurement of the optical fiber bend angle and direction is shown in Figure 2, and it comprises a modified Fused Deposition Modeling 3D printer whose role is to act as a computer-controlled 3-axis translation stage with a 50 μm step resolution. The print head is removed and substituted by the bend-sensitive optical fiber probe head, while the print bed is overlaid with a 1 mm spacing grid mesh in order to be able to determine the optical fiber tip x- and y-axis values. In this case, x and y values refer to the planar coordinates perpendicular to the optical fiber longitudinal axis, and the z-axis is defined as the axis parallel to the bend-sensitive optical fiber length (see coordinate system on the upper left corner of Figure 2). A digital camera is used to capture images sequentially as the optical fiber tip is deformed. The images are used to extract the optical fiber tip deformation values during the experiment. The inscribed FBGs are located at the clamping point of the optical fiber to the probe head with the middle of the 4 mm long FBGs being roughly in the middle of the clamping zone, as determined by the cross-sectional area of an M2 screw (see inset in Figure 2). The optical fiber is protected by a 900 μm outer diameter furcation tube (FT900Y Hytrel furcation tube from Thorlabs, Newton, NJ, USA) with a 400 μm inner diameter, and the total optical fiber length protruding from the probe head is 14.5 mm (distance L in Figure 2). For the given optical fiber protrusion length, the response of the sensor is indifferent to the furcation tubing material, but the latter will affect the force required by the user to bend the fiber. A different optical fiber protrusion length, however, requires a new calibration procedure to determine a new polynomial parameter set, as it will be described later in the text. Fiber Gragg Grating spectra are acquired by a commercially available 4-channel FBG interrogator (FemtoSensing International, Atlanta, GA, USA), and the interface between the 4-core optical fiber and the FBG interrogator is through a 4-port optical fiber fanout (Meisu Optics, Zhongshan, China).

**Figure 3:**
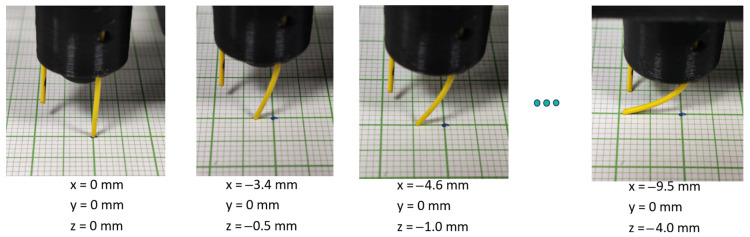
Image sequence of the bend-sensitive optical fiber (optical fiber in the foreground) experimental coordinate determination. On the left side, the initial position is determined (x, y, z = 0, 0, 0), and then the z-axis is sequentially moved towards the grid table, while pictures are taken to determine x- or y-axis coordinates.

The experimental procedure for the optical fiber tip position measurement is as follows: Initially, the optical fiber tip is gradually lowered using a custom computer interface towards the surface of the grid mesh and centered on top of a grid line cross-section (see Figure 3, left). The FBG spectra are continuously monitored during this initial touch-down procedure in order to prevent the optical fiber from deforming; namely, the optical fiber tip is lowered until contact is achieved without any shift to the spectral locations of the 4 individual FBGs in the 4-core optical fiber. The z-axis step resolution towards the final fine-tuning steps is set to 50 μm.

The next step is the parallelization of the probe head with respect to the grid mesh table. This step is important to ensure similar bend behavior—and, in turn, spectral response—towards both positive and negative x- and y-axis direction deformation. The probe head was moved towards predefined z-axis positions (−0.5 mm, −1.0 mm, and −2.0 mm, where the minus sign indicates motion towards the grid mesh table) and the optical fiber tip was manually aligned one time towards the positive x direction; then, the process was repeated towards the negative x direction. Camera images were taken to define the tip x coordinate, and if the coordinates from both directions did not agree in absolute value, the probe head was tilted using a set of alignment screws to correct for the tilt. The same process was repeated on the y-axis until all four coordinate readings were of similar absolute value, indicating a good leveling between the probe head and the grid mesh table.

After the leveling procedure, tip coordinate determination followed, which acts as the ground truth and calibration table for the algorithmic reconstruction. A g-code script was used to move the z-axis towards the grid mesh table at a step of 0.5 mm with a positioning error of 50 μm (0.50 mm step ± 0.05 mm). At each step, the optical fiber was aligned along a predetermined axis direction (x or y), and consequent camera images were taken in order to determine the tip location, as shown in the picture sequence of Figure 3.

**Figure 4:**
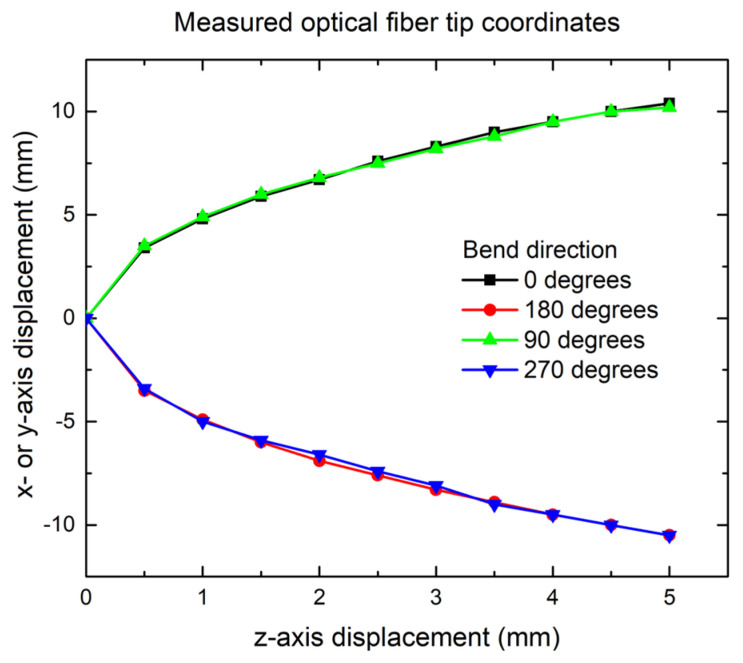
Geometrical space of bend-sensitive optical fiber tip of length L = 14.5 mm as it bends along the x- or y-axis. The tip x- or y-axis displacements overlap for all 4 directions within a 0.05 mm error.

The resulting geometrical space of the tip position for various z-axis displacements is plotted in Figure 4 for four different directions in the x- and y-axes (optical fiber bent in the +x-axis direction is the 0 degrees orientation, +y-direction bend is 90 degrees, −x is 180 degrees, and finally, the −y direction is 270 degrees). It should be noted that these experimentally derived results are unique to this particular combination of optical fiber–furcation tube, and if the furcation tube would be made of a different material or of a different diameter, these results would have to be updated with new measurements. The overlapping results in both the x and y directions and for both positive and negative displacements show an excellent surface leveling procedure.

## 3. Optical Fiber Tip Coordinate Determination Algorithm and Validation

### 3.1. Curvature Vector Calculation

**Figure 5:**
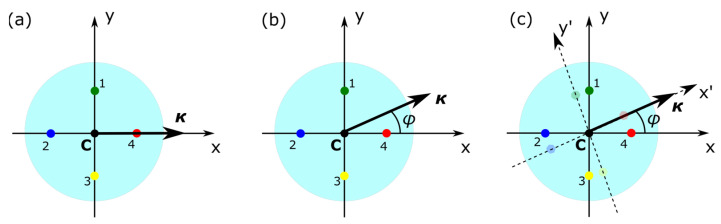
(**a**) Original calibration coordinate system with core #4 aligned to the Cx-axis. (**b**) An arbitrary optical fiber curvature κ at angle φ with respect to the x-axis and (**c**) rotation of the coordinate system by angle φ with respect to the original coordinate system.

The input data from any multi-core Fiber Optic Shape-Sensing system based on FBGs are the wavelength shifts monitored on each FBG during bending, as shown in Figure 1b. For a uniform, first-order FBG, the reflected wavelength *λ_B_* is given by [37]:

(1) $$\lambda_{B}=2n_{eff}\Lambda ,$$

where *n_eff_* is the effective refractive index of the propagating mode, and Λ is the grating periodicity. The wavelength shifts Δ*λ_Β_* on the FBGs due to mechanical strain *ε* exerted on the optical fiber during bending are expressed as [37]:

(2) $${\Delta \lambda }_{B}=\lambda_{B}\left(1-p_{e}\right)\varepsilon ,$$

where *p_e_* is the strain-optic co-efficient, which has a typical value of 0.218 for fused silica-based optical fibers at a wavelength around 1550 nm [38]. Equation (2) is valid if no temperature changes are present, which is an assumption that is undertaken throughout this work, given the temperature-controlled environment of a laboratory (25 °C). The strain value derived from Equation (2) is used to calculate a curvature vector, which provides the spatial deformation of the optical fiber at the location of the Bragg gratings. There are several approaches in composing the curvature vector ***κ*** from the derived strain data, and in this work, the approach established by Moore and Rogge [34], and later followed by Wolf et al. [33], is chosen for its simplicity and generality in symmetrical multi-core optical fibers. According to their work, the curvature vector ***κ*** for a symmetrical optical, with its cores having an equal distance *r* from the optical fiber center, can be expressed as:

(3) $$\kappa =-\frac{2}{Nr}\left(\sum_{i=1}^{N}\varepsilon_{i}cos\theta_{i}n_{x}+\sum_{i=1}^{N}\varepsilon_{i}sin\theta_{i}n_{y}\right)$$

where *N* is the number of optical fiber cores (in this work N = 4), *r* is the core distance from the optical fiber center C (see Figure 1a; in this work, it is 50·√2 μm ≈ 70.7 μm), ***ε_i_*** is the strain calculated for the FBG located on core *i*, θ*_i_* is the angle between the *i*-th core and the unit vector ***n_x_***. Given the fact that the coordinate system was chosen so that the optical fiber cores are aligned with respect to the x- and y-axes, the angles θ*_i_* can be defined as θ_1_ = π/2, θ_2_ = π, θ_3_ = 3π/2, and θ_4_ = 0 (see Figure 5a).

In such a configuration, such as the one depicted in Figure 5a, and under bending of the optical fiber along the x-axis, optical fiber cores 4 and 2 undergo compression and elongation, respectively, giving rise to wavelength changes (and therefore strain changes) larger in magnitude with respect to cores 1 and 3, which undergo deformations of lesser magnitude. If the optical fiber cores are misaligned with respect to the original reference coordinate system and an angle ***φ*** is formed between the cores and the reference system, the angle can be defined using (see Figure 5b):

(4) $$\tan\left(\varphi \right)=\frac{\kappa_{y}}{\kappa_{x}},$$

(5) $$\varphi =atan\left(\frac{\kappa_{y}}{\kappa_{x}}\right)$$

Curvature vector ***κ*** is a two-dimensional vector of the form ***κ*** = (***κ_x_***, ***κ_y_***), and the angle ***φ*** represents the optical fiber rotation with respect to the coordinate system xCy of Figure 5c, where core #4 is aligned to the Cx-axis, forming an angle θ_4_ = 0.

The experimental data of the optical fiber tip position with respect to the probe z-axis proximity to the test table are given in Table 1, along with the calculated magnitude of curvature vector ***κ*** and angle ***φ***.

In Table 1, X_e_, Y_e_, and Z_e_ coordinates are the experimentally observed values of the optical fiber tip, as described in Section 2. Using Equation (5), we calculated the rotation angle ***φ*** and |κ|, the curvature vector magnitude, as derived using Equation (3). The negative values in the Z-axis coordinate denote motion of the probe head towards the test table, and the positive X-axis values denote bending towards the positive x-axis direction (the opposite to that displayed in Figure 3).

### 3.2. Generalization via Exponential Fit

Taking into account the results of Figure 4, it is assumed that the shape of the optical fiber can be expressed by a second-order polynomial function of the form with a satisfactory fit along the full optical fiber length:

(6) $$f\left(x\right)=\alpha_{2}x^{2}+\alpha_{1}x+\alpha_{0}$$

**Figure 6:**
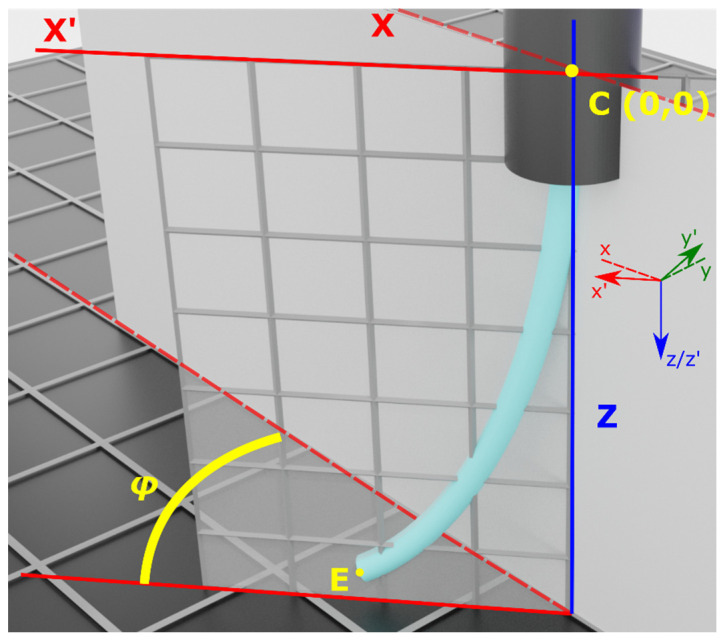
Coordinate system rotation by an angle φ. The original coordinate system is defined by plane zCx, while the rotated coordinate system is defined by plane zCx′. Ultimately, endpoint E coordinates are extracted with respect to the original coordinate system zCx.

Because of the fact that the curve must cross the (x,y) = (0,0) point, the term *α*_0_ = 0. In this case, x corresponds to the Cx-axis coordinate rotated by angle *φ*, or according to Figure 5c, it is the x coordinate along axis Cx’. As a result, the second-order polynomial, calculated according to Equation (6), is located on plane Zx’, rotated in space by an angle ***φ***, as illustrated in Figure 6.

### 3.3. Tip Coordinate Extraction

In order to reconstruct the shape of the optical fiber using the polynomial of Equation (6), the coefficients a_2_ and a_1_ must be determined using the experimental data and in accordance with the curvature vector value.

The curvature vector ***κ*** value is given as input to a system that requires the curve endpoint (E) to coincide with the experimental values, as listed in Table 1 and plotted in Figure 4. In order to achieve curvature matching of the experimentally derived data with the curvature that corresponds to the second-order polynomial, the following condition must apply at point C on plane x’Cz [39]:

(7) κ=f′(1+f′2)32

From Equation (6), the first and second derivatives are calculated as:

(8) $$f^{\prime }\left(x\right)=2\alpha_{2}x+\alpha_{1},$$

(9) $$f^{\prime \prime }(x)=2\alpha_{2}$$

At point C (0,0) as shown on Figure 6, Equations (8) and (9) become, respectively:

(10) $$f^{\prime }\left(0\right)=\alpha_{1},$$

(11) $$f^{\prime \prime }\left(0\right)={2\alpha }_{2},$$

and the relation between the experimentally measured curvature vector κ and the polynomial coefficients becomes:

(12) κ=2a2(1+α12)32

Solving for *α*_2_, Equation (12) becomes:

(13) a2=12κ(1+a12)32

A second condition that is required for the second-order polynomial is to satisfy the endpoint coordinates of the optical fiber tip, as determined by the experiments and presented in Table 1 (X_e_, Y_e_, and Z_e_). By substituting Equations (13) and (6) for the tip coordinates, one gets:

(14) fxe=12κ(1+a12)32 · xe2+α1· xe=ze

Equation (14) can be analytically solved for *α*_1_, and the resulting value can be substituted into Equation (13) to acquire both polynomial coefficient values for each experimentally derived curvature point. Taking into account the fact that the optical fiber length L is known (L = 14.5 mm), it is possible to verify the validity of the derived coefficient pairs by solving for optical fiber length according to the arc length determination formula for second-order polynomials [40]:

(15) $$L=\int_{0}^{x_{e}}\sqrt{1+{{(f}^{\prime }(x))}^{2}}dx$$

The integral is solved for the optical fiber length, namely from x = 0 until x = *x_e_*, the endpoint of the optical fiber. By substituting Equation (8) into Equation (15), one gets:

(16) $$L=\int_{0}^{x_{e}}\sqrt{1+{\left(2\alpha_{2}x+\alpha_{1}\right)}^{2}}dx$$

Alternatively, one can calculate the optical fiber length numerically using:

(17) $$L=\sum_{n=2}^{M}\sqrt{{\left(x_{n}-x_{n-1}\right)}^{2}+{\left({f(x}_{n})-{f(x}_{n-1})\right)}^{2}}\;$$

where M is the number of sample points of x used for the validation from x = 0 until x = *x_e_*.

Table 2 contains the analytically derived polynomial coefficient values *α_1_* and *α_2_* for the experiments presented in Table 1, along with a numerical verification of the optical fiber length L using Equation (17).

**Figure 7:**
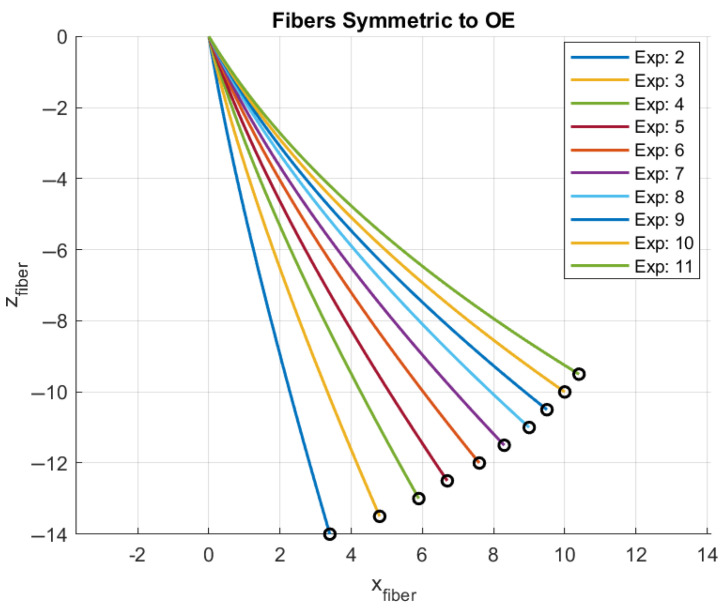
Optical fiber shape reconstruction approximation using 2nd-order polynomial functions.

Using the polynomial coefficients from Table 2, it is possible to reconstruct the optical fiber shape approximation in the z-x plane for the many positions measured using the camera and presented in Figure 4. The corresponding plot is presented in Figure 7.

**Figure 8:**
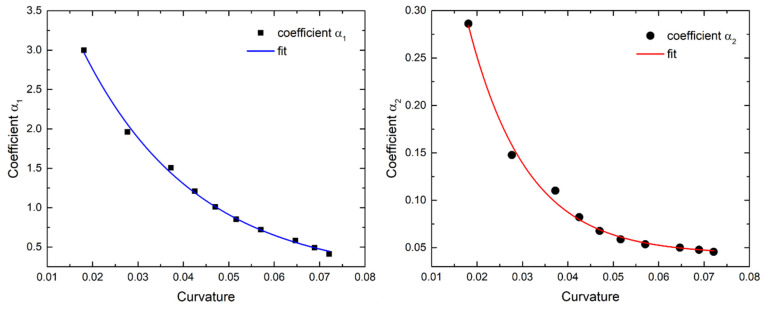
Analytically calculated polynomial coefficient values α_1_ and α_2_ as a function of calculated curvature (solid points). Exponential decay fit of the coefficients (colored solid lines).

The set of second-order polynomial coefficients derived from the experiments is only a very small fraction of actual displacements that can take place during optical fiber bending. Any practical application of the method would require a means to calculate the second-order polynomial coefficients for any arbitrary curvature value. In order to generalize the use of this method, the experimentally derived coefficient values are plotted as a function of optical fiber curvature, as shown in Figure 8.

### 3.4. Exponential Fit Generalization

As can be observed in Figure 8, the distribution of both polynomial coefficients *α*_1_ and *α*_2_ approximates an exponential decay. Such a function was utilized to fit the data with the purpose of acquiring an equation that can be used to extract *α*_1_ and *α*_2_ values for any arbitrary value of curvature that is derived from the Bragg wavelength shifts. The exponential decay fit used is of the form:

(18) $$a_{j=1,2}\left(\kappa \right)=A_{0,j}\cdot e^{-\kappa \cdot t_{0,j}}+y_{0,j},$$

where *A*_0,j_ is the amplitude coefficient, *t*_0,j_ the decay constant, and *y*_0,j_ an offset. The fitting results for *α*_1_ and *α*_2_ values according to Equation (16) are presented in Table 3. It should be noted that the fit quality of the data in both cases exhibited an R^2^ value higher than 99.2%.

The coefficients of Table 3 can be used in Equation (18) to provide the second-degree polynomial coefficients *α_1_* and *α_2_* for any curvature value that is derived from the experimentally measured Bragg wavelength shifts. From there, Equation (16) can be used for the calculation of the X*_e_* coordinate, Equation (14) for the Z*_e_* coordinate, and finally, trigonometry and the value of angle *φ* for Y*_e_*-coordinate determination.

In brief, the overall method can be summarized in two main parts. The first one is the necessary calibration procedure, which is necessary for the acquisition of the polynomial coefficient determination Equation (18). Once this calibration is completed, the optical fiber tip coordinate determination algorithm, namely the second part of the method, can be summarized in the following steps:

1. Measurement of the Bragg wavelength shifts with respect to the initial (resting) position;
2. Calculation of the curvature vector ***κ*** using Equation (3);
3. Calculation of the bend angle direction ***φ*** using Equation (5);
4. Application of Equation (18) along with the fit parameters from Table 3 to determine the polynomial coefficients *α*_1_ and *α*_2_ at curvature ***κ***;
5. Use of Equation (16) to acquire the coordinate X_e_ (numerical solution or analytical approximation);
6. Application of coordinate X_e_ in Equation (14) to acquire coordinate Z_e_;
7. Use of the calculated angle ***φ*** in order to acquire coordinate Y_e_ using trigonometry and the X_e_ coordinate.

## 4. Results

**Figure 9:**
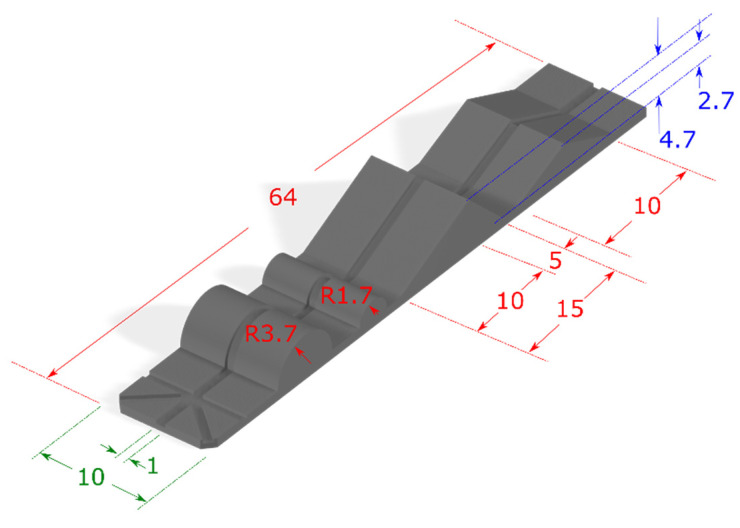
Dimensions (all in mm) and 3D shape of the test structure. The 1 mm wide track in the middle has a depth of 0.4 mm in order to keep the optical fiber tip centered along the path.

The developed algorithm has been tested for accuracy using two different 3D-printed test structures. The geometry of the first test structure is shown in Figure 9.

The geometrical features of this structure include an isosceles and a scalene triangle along with two hemi-cylinders of 1.7 mm and 3.7 mm radii. The maximum height of the two triangles was 4.7 mm and 2.7 mm for the scalene and the isosceles triangle, respectively. In the middle of the structure a 1 mm wide and 0.2 mm deep track was designed in order to prevent the optical fiber tip from sliding along the lateral direction.

**Figure 10:**
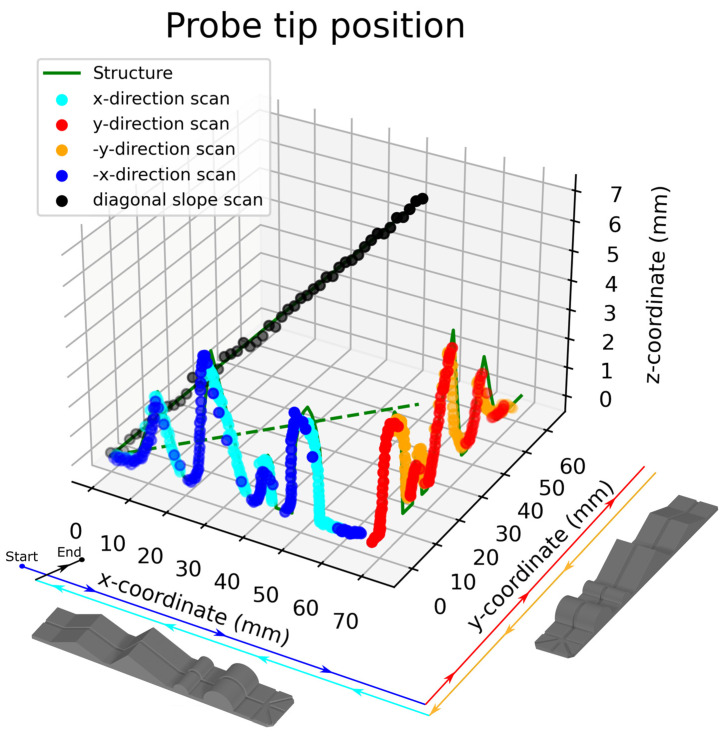
Probe tip coordinate measurement (blue, cyan, orange, red, and black dots) versus real shape coordinates (green line). The dashed green line is the x, y-axis projection of the diagonal scan. The structure orientation is presented below the x- and y-axes along with scan trajectory.

Two multiples of the test structure were fabricated and placed on the setup test bed at 0- and 90-degree angles in order to test the response of the sensor under different directions in space. The testing procedure was automated and included steps of 0.5 mm in either the x or y direction and the automatic, real-time plotting of the optical fiber tip position. Both positive and negative x- and y-axis directions were scanned in order to cover all features of the structure. Additionally, a structure with a linear slope and maximum height of 7 mm was placed diagonally at the start point of the scan to demonstrate an example with variations in all three axes at the same time. The optical fiber tip endpoint coordinates are plotted against the structure coordinates in the three-dimensional plot of Figure 10. Solid lines represent the structure coordinates (ground truth), and dots represent the optical fiber tip coordinates, as calculated by the proposed algorithm. The dashed green line is the x- and y-axis projection of the diagonal slope scan (black dots).

The x, y, and z coordinates of the optical fiber probe tip, as calculated by the proposed method, are plotted as cyan and red dots for the scanning along the positive x- and y-axis directions and blue and orange for the negative ones, as indicated in Figure 10. Both directions were scanned in order to compensate for the optical fiber tip leaps after reaching the peak position of a structure. Additionally, scanning an axis along both directions can reveal possible calibration errors or leveling, therefore acting as a calibration verification method. All measurements were realized with a 0.5 mm step along the x- or y-axis. As presented in Figure 10, the optical fiber tip follows the target structure with an accuracy that was determined to be better than 2% in x-axis and 1% in the lateral axes (x or y, depending on direction). The lateral displacement of the optical fiber tip was prevented by the use of an embossed track in the middle of the target structure, and therefore, the calculated error in this direction was better than 1% (maximum displacement of 0.5 mm over a length of 70 mm).

**Figure 11:**
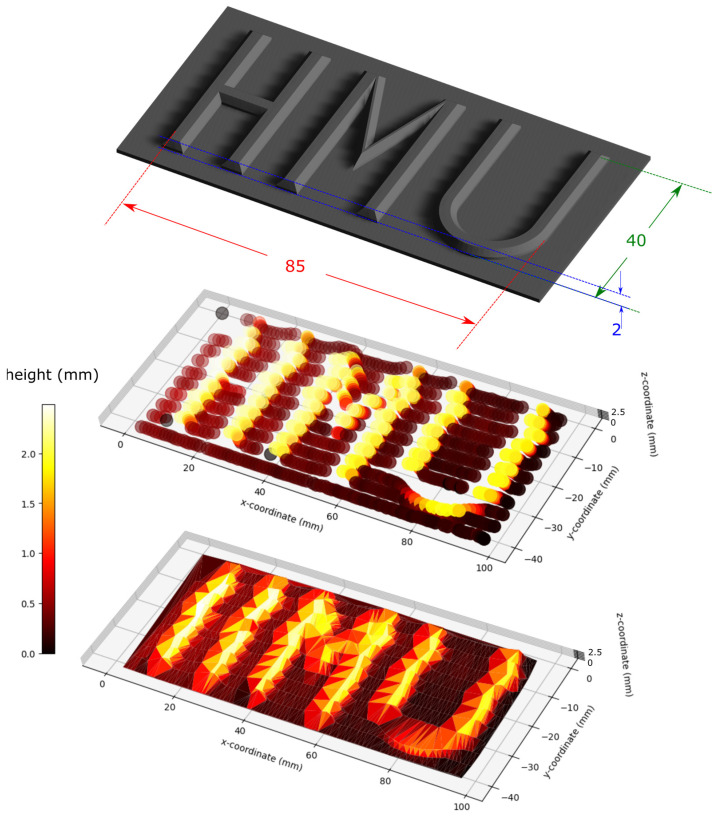
The 3D reconstruction of a solid structure by line scanning and using the optical fiber tip coordinates. Top: solid structure as designed and 3D printed. Middle: line scan results of the optical fiber tip scan. Bottom: shape reconstruction by utilizing coordinate set triangulation. HMU initials stand for Hellenic Mediterranean University.

The developed algorithm was then used to line scan a 3D-printed structure with the initials of HMU, i.e., Hellenic Mediterranean University, with a letter height of 2 mm and feature width of 4 mm. A sketch of the structure is presented on the top side of Figure 11.

A total of 11 lines were scanned, and the resulting 11-line plot of the acquired x, y, and z coordinates is presented in the middle plot of Figure 11. It is worth noting that the optical fiber tip could not climb up the curved part of letter ‘U’ but slid along the curvature, which is manifested by a change in the y-axis position coordinate and a smaller height (z-axis). A similar situation took place while scanning the horizontal line of letter ‘H’, where the optical fiber tip slid towards the lower part of this feature. Overall, the method was capable to satisfactorily create a 3D plot of the structure with only 11 lines of resolution along the structure width.

## 5. Discussion

The results presented in this study validate the proposed polynomial-based tip-positioning algorithm as a robust and computationally efficient alternative to traditional FBG-based shape reconstruction methods. The approach accurately estimates the 3D tip position of a multi-core optical fiber using only local Bragg wavelength shifts and a curvature-to-polynomial mapping derived from a single calibration procedure.

Compared to more complex shape reconstruction techniques based on differential geometry, transformation matrices, or stochastic estimation, the proposed method significantly reduces computational overheads. A modern microcontroller is sufficiently powerful to provide the necessary computing power for the calculations, and the method can be fully covered by an analytical solution if the solution of Equation (16) is approximated using an analytical solution.

Methods involving full curve integration are often iterative and sensitive to cumulative errors, particularly for short fiber segments. In contrast, our technique avoids such propagation by directly relating curvature to a low-order polynomial model, achieving high accuracy with minimal processing demands. Additionally, unlike approaches that require external tracking systems during operation, our method achieves accurate reconstruction using only initial ground-truth measurements. The accuracy levels achieved—under 2.5% in all directions and sub-millimeter absolute errors—are competitive with or superior to the existing literature benchmarks for short fiber lengths. In this work, however, due to the small optical fiber protrusion length of 14.5 mm, optical fiber twist was considered as negligible and was not taken into account. In future work, the authors intend to employ a birefringent FBG [41] in one of the optical fiber cores that can compensate locally for optical fiber twist by monitoring the relative distance between the split peaks of the birefringent FBG.

All of the experiments described in this work were undertaken under laboratory conditions, namely at a temperature of 25 °C and a relative humidity ranging from 40 to 60%. Although optical fibers are not inherently sensitive to humidity, temperature variations, however, can affect the accuracy of the sensing device. Typically, temperature is either compensated by a compensation core in the middle or by employing a temperature compensating single-core optical fiber alongside the path of the shape-sensing optical fiber.

In the case of use in harsh environments, the protective furcation tubing of the multi-core optical fiber can be substituted by a stronger tube or even metallic capillary. As already mentioned, the type of protection around the optical fiber does not affect the output of the method but only affects the amount of force required to bend the optical fiber. The furcation material can be chosen as a compromise between sensor endurance (optical fiber will break under a certain bend angle) and force required to bend the sensor. In its current form, the optical fiber was bent at angles up to 100° without failure.

**Figure 12:**
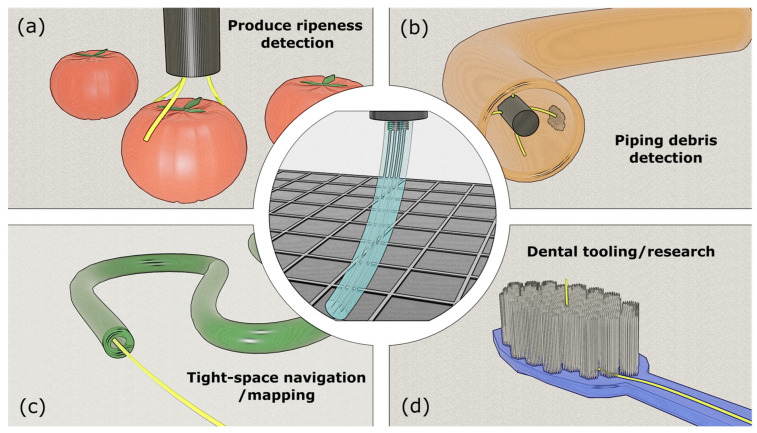
Applications of tip-positioning optical fiber sensor (middle): (**a**) fruit/vegetable ripeness sensor, (**b**) piping debris/agglomeration detection, (**c**) tight-space navigation/mapping, and (**d**) dental tooling/research.

Given its lightweight computational load and compact sensor footprint, this method is particularly well suited in domains such as the following: medical navigation: integration into minimally invasive surgical tools, where real-time tip feedback is essential, and space constraints are severe; robotics and soft actuators: real-time control of soft continuum robots and end effectors; inspection and mapping: low-power deployment in pipeline inspection, structural scanning, and confined space exploration; and agricultural and food tech: insertion-based testing (e.g., fruit ripeness or internal defect detection), where tactile sensing is advantageous. These are summarized in Figure 12.

The method also lends itself to applications requiring repeated or automated scanning, as shown with the 3D-printed letter test case. This opens up possibilities for simple yet robust surface profiling systems.

However, the method, despite its merits, inherits some intrinsic error sources related to the nature of the FBG sensor itself. Primarily, throughout the length of this work it is assumed that the temperature during measurements remains constant, which might be true for some applications, but temperature compensation will be required for some others where temperature variations occur. Additionally, the method is relying on an initial calibration stage, where careful optical fiber alignment and leveling with respect to the surface it bends on has to take place; otherwise, systematic errors will propagate across all calculations.

Nevertheless, a precise calibration procedure can result in very good performance results, which are summarized on Table 4.

## 6. Conclusions

This work introduces a novel algorithm for estimating the 3D tip position of a bent multi-core optical fiber with embedded Fiber Bragg Gratings (FBGs), using a second-order polynomial shape approximation and a curvature-to-coefficient mapping derived from a one-time calibration. The method eliminates the need for complex curve integration or external tracking systems, offering real-time, sub-millimeter accuracy with minimal computational load.

The algorithm was validated using precision-controlled experiments and real-world 3D-printed test structures, where it achieved positional errors below 2.5% in all spatial directions. Its lightweight implementation and low power requirements make it ideally suited for edge-computing applications, including medical navigation, robotic actuation, structural inspection, and soft material interaction.

Future work will focus on generalizing the method to capture torsion and full 3D curvature, incorporating temperature compensation, and integrating the algorithm into embedded systems for field deployment, as well as twist compensation in the form of a birefringent FBG in one of the optical fiber cores.

## Figures and Tables

**Table 1:** Optical fiber tip coordinates and calculated curvature vector and angle φ values.

X_e_	Y_e_	Z_e_	φ		|κ|
mm	mm	mm	rad	°	mm^−1^
0	0	0	-	-	0
3.5	0	−0.5	0.095	5.43	0.022
5	0	−1.0	0.072	4.14	0.031
6	0	−1.5	0.099	5.72	0.038
7	0	−2.0	0.088	5.05	0.045
7.8	0	−2.5	0.082	4.71	0.050
8.5	0	−3.0	0.063	3.61	0.057
9	0	−3.5	0.049	2.81	0.061
9.5	0	−4.0	0.057	3.27	0.066
10	0	−4.5	0.047	2.68	0.068
10.5	0	−5.0	0.051	2.93	0.073

**Table 2:** Analytically derived polynomial coefficient values α_1_ and α_2_, along with optical fiber length L verification.

X_e_	Y_e_	Z_e_	φ	|κ|	α_1_	α_2_	L
mm	mm	mm	°	mm^−1^			mm
0	0	0	-	0	-	-	14.50
3.5	0	−0.5	5.43	0.022	2.89	0.32	14.44
5	0	−1.0	4.14	0.031	1.91	0.16	14.42
6	0	−1.5	5.72	0.038	1.50	0.11	14.35
7	0	−2.0	5.05	0.045	1.19	0.08	14.38
7.8	0	−2.5	4.71	0.050	0.99	0.07	14.38
8.5	0	−3.0	3.61	0.057	0.82	0.06	14.39
9	0	−3.5	2.81	0.061	0.71	0.06	14.32
9.5	0	−4.0	3.27	0.066	0.60	0.05	14.29
10	0	−4.5	2.68	0.068	0.51	0.05	14.29
10.5	0	−5.0	2.93	0.073	0.41	0.04	14.34

**Table 3:** Exponential decay fit coefficients.

Polynomial Coefficient	A_0_ Value	t_0_ Value	y_0_ Value
α_1_	6.914	43.05	0.189
α_2_	1.950	90.09	0.048

**Table 4:** Method performance summary.

Structure	Structure Feature	Max Height (mm)	Measured Height (mm)	Tip Error %
Test structure 1	Circle 1	3.5	3.44	1.6
	Circle 2	1.5	1.46	2.4
	Triangle 1	4.5	4.40	2.0
	Triangle 2	2.5	2.43	2.5
HMU letters	Mixed	2.0	1.93–1.97	<2.5
	‘H’—horizontal line	2.0	missed	
	‘U’—curved part	1.76 (fiber bent)	1.73	1.7

## Data Availability

Data underlying the results presented in this paper are not publicly available at this time but may be obtained from the authors upon reasonable request.

## Abbreviations

The following abbreviations are used in this manuscript:

CSS	Conventional Shape Sensor
FOSS	Fiber Optic Shape Sensor
FBG	Fiber Bragg Grating
